# A green, efficient and precise hydrogen therapy of cancer based on *in vivo* electrochemistry

**DOI:** 10.1093/nsr/nwz199

**Published:** 2019-12-05

**Authors:** Guohua Qi, Bo Wang, Xiangfu Song, Haijuan Li, Yongdong Jin

**Affiliations:** 1 State Key Laboratory of Electroanalytical Chemistry, Changchun Institute of Applied Chemistry, Chinese Academy of Sciences, Changchun 130022, China; 2 University of Chinese Academy of Sciences, Beijing 100049, China; 3 School of Public Health, Jilin University, Changchun 130021, China

**Keywords:** hydrogen therapy of cancer, *in vivo* electrochemistry, acupuncture Fe needle

## Abstract

By combined use of traditional Chinese acupuncture Fe needle electrode and *in vivo* electrochemistry, we achieved *in vivo* H_2_ generation in tumors in a controllable manner and exploited it for effective and green therapy of tumors for the first time. The cathodic acupuncture electrodes working under an applied voltage of ∼3 V (with minimal damage to the living body) undergo effective electrochemical reactions in the acidic tumor area that produce sufficient H_2_ locally to cause cancer cells to burst and die. Due to puncture positioning, the acidic tumor microenvironment and gas diffusion effect, the developed H_2_ generation electrochemotherapy (H_2_-ECT) strategy enables precise and large-scale tumor therapy, as demonstrated by *in vivo* treatment of diseased mice (glioma and breast cancers). Such green H_2_-ECT is simple, highly efficient and minimally invasive, requiring no expensive medical equipment or nano materials and medication, and is therefore very promising for potential clinical applications.

## INTRODUCTION

Up to now, cancer is still one of the major diseases that threaten the survival of mankind, and it is difficult to cure clinically [1]. In addition to single or combined surgery, chemotherapy and radiotherapy, which are commonly used clinically [2], a number of promising therapeutic strategies have been recently put forward including immunotherapy and gene therapy, photothermal therapy (PTT), photodynamic therapy (PDT), and so on [3–10]. However, these techniques usually rely on chemical and genetic drugs or exotic nanomaterials to actualize treatments, making them quite difficult or debatable for practical clinical applications in the near future due to the uncertainties of potential biotoxicity and biohazards, and related genetic and ethical issues [11,12]. Also, immunotherapy and gene therapy are complex and expensive [13,14]. Therefore, the popularization of these techniques in clinical practice is restricted. Consequently, the development of simple, green, efficient and cheap treatment methods is urgently needed to combat cancer.

Hydrogen (H_2_), owing to its small molecular size and physiological inertness, resistance to oxidation, and good gas diffusivity *in vivo*, is considered as a kind of green and endogenous gas [15]. H_2_ can easily penetrate into the biological membrane to disperse into cytoplasm and other organelles such as the nucleus, mitochondria and so on due to its small size. It performs eminent physiological/pathological regulatory functions, and this has been exploited for the treatment of many diseases, such as Alzheimer's disease, arthritis and diabetes [16–19]. In 2007, Shigeo Ohta *et al.* demonstrated that the use of H_2_ as a therapeutic antioxidant can selectively reduce cytotoxic oxygen radicals [16]. Several reports have also confirmed that a low concentration of H_2_ has therapeutic effects against local inflammation such as eye, ear, nose, liver and systemic inflammation [20–24]. Dole *et al.* attempted to use the antioxidation ability of hyperbaric H_2_ to treat skin squamous cell carcinoma in 1975 [25], but the need to provide hyperbaric H_2_ with diving medical equipment limited the possible clinical application of hyperbaric H_2_ in tumor therapy. Subsequently, to obtain a high

concentration of H_2_, the conventional routes of H_2_ administration are to inhale H_2_ gas and intake of H_2_-rich water or saline [22,23,26]. These approaches, due to the lack of targeting for the tumor, along with poor bioavailability caused by poor water solubility of H_2_, limited their therapeutic efficacy. More recently, Zhao *et al.* tried using near-infrared (NIR) light-responsive PdH_0.2_ nanocrystals as carriers to deliver H_2_ and realize synergistic therapy of photothermal and H_2_ therapy [27]. However, it is still difficult to achieve sufficient H_2_ release *in vivo* with this method and it has potential biotoxicity due to the introduction of nanoparticles. To the best of our knowledge, no successful cancer therapy by the exploitation of H_2_ alone has been reported. In fact, how to produce H_2_ non-invasively and sufficiently without using nanomaterials and how

to achieve the release of H_2_ on demand *in vivo* are two huge challenges facing the H_2_ therapy of cancers.

**Figure 1.**
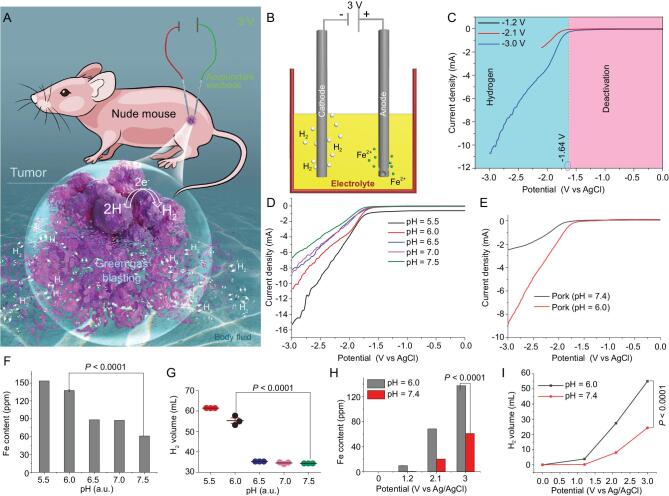
(A) Schematic diagram of green H_2_-ECT therapy *in vivo*. (B) Schematic EC processes of two acupuncture Fe electrode systems showing anodic Fe dissolution and cathodic H_2_ production. (C–E) The H_2_-production polarization curves of acupuncture electrodes recorded, respectively, in simulated body fluid (SBF, pH = 6.0) under three different voltages (C), in SBF but with different pH (5.5, 6.0, 6.5, 7.0 and 7.5) at 3 V (D), and in fresh pork (mimicking complex tumor tissue) at two pHs (6.0 and 7.4) (E). (F) Dissolved amount of iron from the anode during the EC process. (G) The dependence of H_2_ production of the system on various pH levels in SBF recorded at 3 V for 10 min (*n* = 3). (H, I) The dependence of the amounts of dissolved iron and produced H_2_ on voltages varying from 0 to 3 V, each for 10 min duration, at pH = 6.0 and 7.4, respectively (*n* = 3). All the data are represented as mean ± SEM (standard error of the mean). *n* denotes samples or number of experiments. *n* = 3, *P* < 0.0001 representing the statistical significance of different pH values (pH = 7.5 and pH = 6.0), which is evaluated by two-tailed Student's t-test with GraphPad Prism software.

Acupuncture is a traditional and unique minimally invasive method to treat diseases in China. It is quite effective for the treatment of systemic diseases, especially arthritis, cervical spondylopathy, psoatic strain and so on [28,29], but applying it to the treatment of major diseases, such as cancers, is still a great challenge. In this study, by innovative combined use of the traditional Chinese acupuncture Fe needle (electrode) and *in vivo* electrochemistry, we developed a simple and precise cancer therapy approach based on selective electrochemical generation of H_2_ in the tumor region, termed *in vivo* H_2_ generation electrochemotherapy (H_2_-ECT). Very impressively and excitingly, as depicted in Fig. 1A, the two acupuncture electrodes inserted into the tumor lump work well at ∼3 V direct current (DC) electric field (which causes minimal damage to healthy tissues), driving the electrochemical H_2_ generation reaction effectively under the acidic tumor microenvironment and destroys solid tumors. Importantly, the H_2_-ECT method enables large-scale tumor therapy by applying a gas diffusion effect, avoiding the shortcoming of limited effective area for classic electrochemical reactions. Moreover, due to the puncture positioning and acidic tumor microenvironment (compared to normal tissue), the method provides ideal selectivity and targeting to precisely kill tumors with minimal damage to normal tissue, which is very promising for potential clinical applications. The effectiveness of the method has been demonstrated by successful and fast treatment of glioma and breast cancers in mice in this study.

## RESULTS

### Characterization of H_2_ generation *in vitro* by the H_2_-ECT method

To verify the production of H_2_ during the *in vivo* electrochemical (EC) process, we first carried out *in vitro* EC studies by using two medical acupuncture needles, whose main component is stainless steel, as cathode and anode electrodes. Figure 1B depicts the two-electrode system used in the experiments. The whole EC reactions are carried out at the electrode/electrolyte interfaces under an applied DC electric field and the simulated body fluid (SBF, pH = 6.0), in which the H_2_ evolution reaction and the acupuncture electrode corrosion process were carried out at the cathode and anode, respectively. The reaction equations are as follows:

$$\begin{equation*}\begin{array}{@{}*{1}{c}@{}}
{{\rm{Cathode}}\,{\rm{reaction}}:2{{\rm{H}}^ + } + 2{{\rm{e}}^ - }\ \to \ {{\rm{H}}_2};}\\
{{\rm{Anode}}\,{\rm{reaction}}:{\rm{ }}{\rm{Fe}} - {\rm{ }}2{{\rm{e}}^ - }\ \to \ {\rm{F}}{{\rm{e}}^2}^ + .} \end{array}\end{equation*}$$

Figure 1C shows typical polarization curves of the cathode in the SBF under three different voltages. H_2_ is gradually generated during the EC process when the cathode voltage is greater than ∼−1.64 V. The known half-reaction standard electrode potentials (*E*^0^) for Fe/Fe^2+^, Fe/Fe^3+^ and Fe^2+^/Fe^3+^ are 0.44, 0.036 and −0.77 V, respectively, relative to the standard electrode potential of H_2_ [30–32]. In this study, the overpotential of acupuncture electrode to produce H_2_ was about 1.2 V, which is much higher than the *E*^0^ of Fe/Fe^2+^ for iron dissolution. The overpotential is caused by the surface passivation of iron in a weakly acidic solution [33,34]. When cathode potential exceeded the passivation potential, the anode electrolysis was increased (Fig. S1 in the online supplementary material). Under the EC conditions, the anode and cathode produced ferrous iron (Fe^2+^) and H_2_, respectively, affirmed by X-ray photoelectron spectroscopy (XPS) and methyl blue (MB) probe UV–Vis spectrum characterizations (Figs S2 and S3). The pH effect of media on the H_2_ production of the system was also checked by recording the polarization curves of the electrodes in SBF with various pH values. The results showed that the more acidic the solution, the more H_2_ was produced (Fig. 1D). The result is the same when using fresh pork (to simulate complex tumor tissue) of two pH values (Fig. 1E and Fig. S4B), which is favorable for potential H_2_-ECT applications *in vivo* since tumor tissue is weakly acidic compared to neutral healthy tissue. To facilitate the evaluation of the therapeutic efficacy of the method, we measured the iron consumption of the acupuncture electrode semiquantitatively using inductively coupled plasma mass spectrometry (ICP-MS), and calculated the volume of H_2_ generation under the various conditions (see online supplementary material for detailed calculation).

As shown in Fig. 1F and G, the iron consumption of the acupuncture electrode and the volume of H_2_ generated were gradually decreased as the pH of SBF increased from 5.5 to 7.5 during the 3 V electrolytic process. We then examined the amount of iron consumption and H_2_ generation volume at pH 7.4 and 6.0, respectively, under three different applied voltages (0, 1.2, 2.1 and 3.0 V) each for 10 min duration. The results indicated that at the two pH levels the volume of H_2_ generated was rapidly increased as voltage increased, especially at pH 6.0 (Fig. 1H and I). This dependency and flexibility of H_2_ generation on pH and voltage make the method promising and operational in a controlled manner for precise H_2_ therapy of cancer *in vivo*.

Before applying the method to *in vivo* cancer therapy, we assessed the potential cytotoxicity of the dissolved Fe^2+^*in vitro* by live/dead staining and MTT assay, using MCF-7 and C6 cells as models. The cells were treated with different concentrations of Fe^2+^ (from 5 μM to 50 mM). As shown in Fig. S5, the cytotoxicity of Fe^2+^ with concentrations less than 5 mM was negligible as iron is an essential nutrient and the most abundant transition metal in the human body [35]. In our system, since the maximum concentration of Fe^2+^ produced by 10 min electrolysis of the electrode at the applied voltage of 3 V (at pH 6.0) was estimated to be about 2.5 μM, the biotoxicity of Fe^2+^ is not a concern. For safety, before cancer treatment *in vivo*, we also checked the injury degree of the H_2_-ECT method for healthy tissue versus cancer tissue, by using the fresh pork immersed in SBF with pH 7.4 and 6.0, respectively, as mimics. As expected, the injury degree of tissue at pH 6.0 was more serious than that on the neutral tissue (Fig. S6A), implying the effectiveness of the method for cancer therapy *in vivo*. We also performed an additional hematoxylin and eosin (H&E) staining assay to further accurately discriminate the injury degree of tissue at different pH levels (pH = 6.0 and pH = 7.4). As shown in Fig. S6B, degeneration and necrosis of the muscle fibers of cytoplasm were observed in the tissue at pH = 6.0 after H_2_-ECT, indicating that cells were seriously damaged. However, after the H_2_-ECT, the muscle fibers were still intact for the tissue at pH = 7.4, which indicated that this method is basically harmless to normal tissue. Consequently, the H_2_-ECT has great medical value in the treatment of cancer.

### Characterizations of H_2_ generation *in vivo* during the H_2_-ECT process

**Figure 2.**
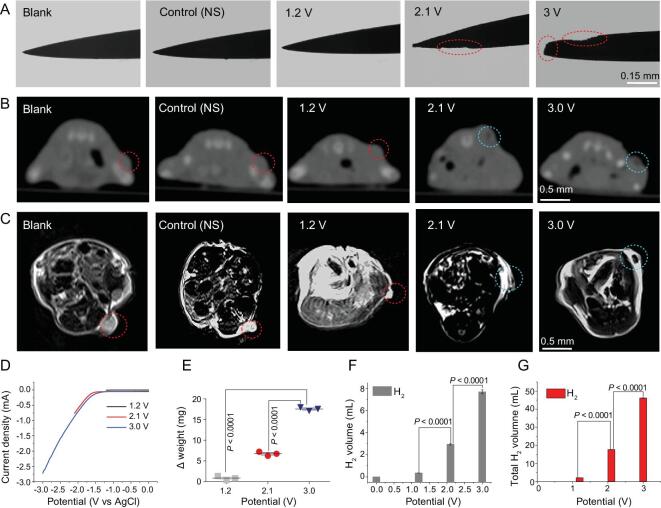
(A) The microscopic imaging of the acupuncture anode electrode after the H_2_-ECT treatment under the various voltages for 10 min. The red circles indicate the corrosion location of the electrode. (B, C) *In vivo* CT and T_1_-weighted MRI images of the C6 tumor-bearing mouse in the different groups (blank, control (needle stimulation (NS) + 0 V), NS + 1.2 V, NS + 2.1 V and NS + 3.0 V). The red circles indicate the tumor sites and the blue circles indicate the tumor sites filling with gas (cavity). (D) The *in vivo* H_2_-generation polarization curves of the acupuncture electrode during H_2_-ECT under three applied voltages for the C6 tumor-bearing mouse. (E, F) The weight loss (E) and volume increase (F) of H_2_ produced from the single electrode in one H_2_-ECT treatment under the different voltages for 10 min (*n* = 3). (G) The calculated total volume of H_2_ generated *in vivo* in the whole H_2_-ECT process under the different applied voltages (0, 1.2, 2.1 and 3.0 V) with a total treatment time of 60 min (10 min twice a day for 3 days) (*n* = 3). All the data are represented as mean ± SEM. *n* denotes the number of mice. *n* = 3, *P* < 0.0001 representing statistical significance of different voltages (1.2, 2.1 and 3.0 V), which is evaluated by two-tailed Student's t-test with GraphPad Prism software.

Firstly, for better observing the therapeutic effect, the tumor-bearing BALB/c mice with similar average tumor size were divided into different groups before H_2_-ECT (Fig. S7). To assess H_2_ production efficiency *in vivo* during the H_2_-ECT process, two closely spaced acupuncture electrodes were inserted carefully and precisely with the naked eyes into the tumor lump of a C6 tumor-bearing mouse and the depth of insertion of the electrodes was decided by the depth and thickness of the tumor. The part of the electrode outside the tumor was insulated by encasing with plastic hose to avoid short circuits and unnecessary damage to normal tissue (Fig. S8). As shown in Fig. 2A, the acupuncture electrodes were gradually electrolyzed with increasing voltages applied from 0 to 3 V, with some surface corrosion observed by microscope after the *in vivo* treatment, indicating the occurrence of the EC reactions. The voltage-related H_2_ production in nude mouse solid tumors was further evaluated by *in vivo* T1-weighted magnetic resonance imaging (MRI) and computed tomography (CT) imaging. As clearly shown in Fig. 2B and C, as the treatment voltage became higher than 2.1 V, cavities emerged in the tumors, indicating effective gas (H_2_) production within the tumor after the H_2_-ECT treatment. Moreover, with the increase of voltage, the cavity area increased gradually, confirming the effectiveness and flexibility of the method for cancer therapy *in vivo*.

Figure 2D shows the *in vivo* H_2_-generation polarization curves of the cathode Fe electrodes recorded inside the tumor in a three-electrode system (with the AgCl-treated Ag puncture electrode as the reference electrode), under three different applied voltages. The result is quite similar to the *in vitro* curves in SBF (pH = 6, cf. Fig. 1C), indicating effective occurrence of the EC reactions in the acidic tumor microenvironment (which provides sufficient protons for the reaction). We further checked electrogravimetric loss (Fe dissolution) of the anode after the H_2_-ECT treatment (Fig. 9). The electrogravimetric loss of the electrode increases with increasing voltage, illustrating increased iron consumption (Fig. 2E). Then, the average amount of H_2_ produced by a single cathode electrode during each 10-min treatment session can be calculated electrochemically according to the above-mentioned equations, to be ∼0.35, 2.95 and 7.69 mL under the applied voltages of 1.2, 2.1 and 3.0 V, respectively. Therefore, in the whole H_2_-ECT process (10 min twice a day for 3 days), the total amount of H_2_ produced in a diseased mouse at a corresponding voltage was ∼2.1, 17.7 and 46.1 mL, respectively. The calculated results are consistent with that obtained by Faraday's law of electrolysis (Fig. S10). These results also agreed well with the electrogravimetric analysis. For further safe tumor therapy *in vivo*, dynamic processes of H_2_ generation and Fe consumption of the electrodes during the *in vivo* H_2_-ECT treatment were monitored (Fig. S11) and the results showed that the amounts of H_2_ generation and Fe consumption tended to stop increasing after the electrolysis time reached more than 10 min during one treatment (Fig. S11B). The reason is that limited tumor local electrolytes near the electrodes will be depleted during one rushing EC run due to the diffusion limitation. In addition, locally growing H_2_ bubbles will increase the EC resistance and slow down the reaction. Fortunately, such self-protection mechanism further reduces the damage to healthy tissue, and these can be easily addressed by intermittent H_2_-ECT or multi-needle treatment (especially for large tumors) to improve therapeutic effects. Therefore, 10 min per treatment is safe and effective and will be used as an optimal time in the following studies.

### Evaluation of the therapeutic effect of H_2_-ECT *in vivo*

**Figure 3.**
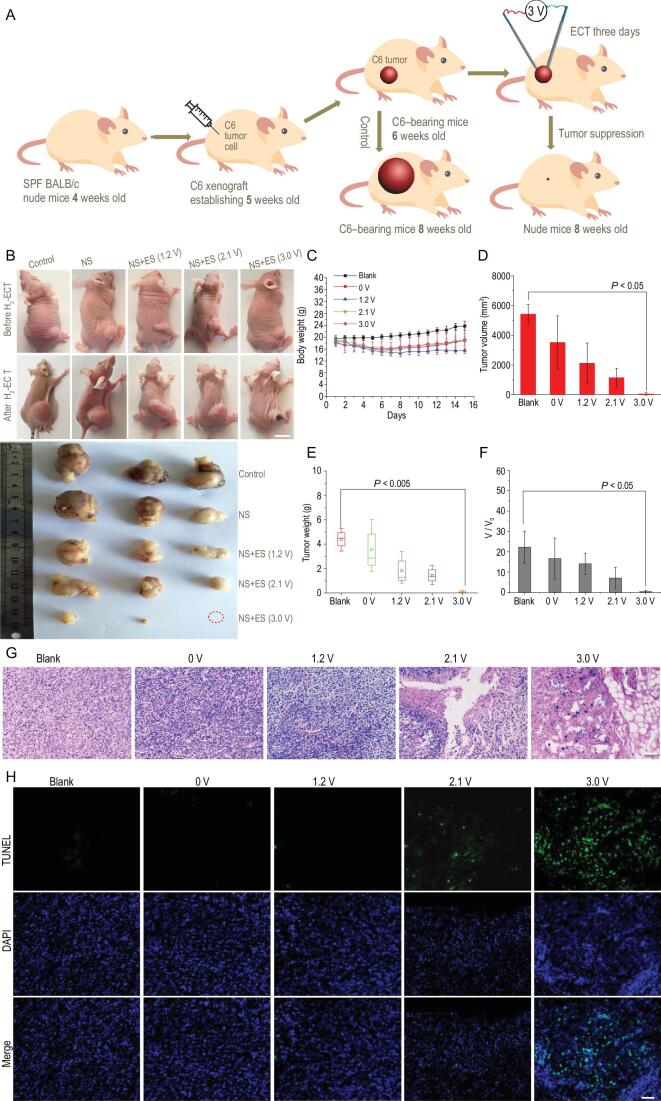
(A) Schematic illustration of C6 tumor xenograft establishment, blank and H_2_-ECT procedures, and therapeutic outcome (*n* = 3). (B) Typical photographs of the tumor-bearing mice and tumor lumps, and their control groups, before and after 15 days of first treatment in different conditions (10 min twice a day for first 3 days). Red circle indicates the eliminated tumor in this case. Scale bar: 1.5 cm. (C) The body weights of C6 tumor-bearing nude mice with different groups, recorded every day. (D–F) Tumor weight and tumor volume dissected and relative tumor volume after 15 days of first treatment in different conditions (10 min twice a day for first 3 days, *n* = 3). All the data are represented as mean ± SEM. *n* denotes the number of mice. *n* = 3, *P* < 0.05 and *P* < 0.005 representing statistical significance, which is evaluated by Student's two-sided t-test with GraphPad Prism software compared to the control group. (G, H) Staining of tumor sections, H&E (G) and TUNEL (H), after 15 days of first treatment under the different groups (blank, NS + 0 V, NS + 1.2 V, NS + 2.1 V and NS + 3.0 V). Scale bar: 50 μm.

The developed H_2_-ECT approach was then exploited for *in vivo* treatment of glioma in diseased mice as it is one of the deadliest cancers and difficult to cure, with a 5-year survival rate of patients less than 5% [36] due to the strongest malignant degree. Consequently, the C6 tumor-bearing BALB/c nude mice were used as a tumor xenograft model to evaluate the *in vivo* tumor therapy capability of the H_2_-ECT method. The control groups and therapeutic groups were administrated to assess the therapeutic performances, respectively (Fig. 3A). Tumor-bearing mice with similar average tumor size were randomly divided into five groups: the blank, needle stimulation (NS + 0 V), NS + 1.2 V, NS + 2.1 V and NS + 3.0 V groups. Although the establishment of the tumor model and feeding of the mice were exactly under the same conditions, there was some obvious volume variation of tumor lumps in the same group due to the individual differences between mice. When the tumor size reached ∼200 mm^3^, each tumor-bearing mouse was treated for 10 min a time twice a day for 3 days using H_2_-ECT and then observed for 12 days, as shown in Fig. S8. The tumor sizes and body weights of the mice were recorded every day. As shown in Fig. 3B, D and E, C6 tumor growth could be effectively inhibited after the H_2_-ECT treatment as the dose of voltage was boosted. In addition, compared to the control groups (blank and NS + 0 V) and therapeutic groups (1.2 V, 2.1 V and 3.0 V), the NS + 3.0 V group presented a significant suppression effect, which is attributed to the highest H_2_ production of the group. The suppression rate in terms of relative tumor volume has been calculated to be 22.19, 16.59, 14.04, 7.03 and 0.38, respectively, for the all tested groups (Fig. 3F). During the 3-day treatment and subsequent 12-day physical recovery, the body weights of mice in control and all therapeutic groups showed no significant changes (Fig. 3C), manifesting the low systemic toxicity of the H_2_-ECT method. Although scab of the treatment area was observed after the H_2_-ECT treatment at 3.0 V, it recovered very well during the recovery process, as shown in Fig. S12. Impressively, the NS + 3.0 V group had the H_2_-ECT treatment conducted only for the first 3 days, but maintained remarkable tumor inhibition effect during the following 12-day observation, indicating that H_2_-ECT is a simple and effective method for tumor therapy. Simultaneously, we also compared the therapeutic process under the 1.2 and 2.1 V treatments and found that the volumes of tumor showed no significant change in the first 4 days after the treatment, but the volumes of the tumor increased gradually during the following recovery process. It implied that insufficient dosage of H_2_ generated during the whole H_2_-ECT process may lead to the rebound of suppressed tumor growth (Fig. S12)

To gain insights into the therapy efficacy, tumor tissues of mice in the different groups were collected and analyzed by hematoxylin and eosin (H&E) staining assays. As shown in Fig. 3G, clear deformation and shrinking of the nuclei and destruction of the membrane integrity were observed in the NS + 3.0 V and NS + 2.1 V groups, indicating that tumor cells were seriously damaged. The tumors of the other groups had no distinct injury. To further confirm the destruction of the tumor by this method, the tumors were sliced after H_2_-ECT and stained using the immunofluorescence terminal deoxynucleotidyl transferase-mediated dUTP-biotin nick end labeling (TUNEL). As clearly seen from Fig. 3H, an intensive green fluorescence, a characteristic of apoptotic signal caused by DNA fragmentation, was observed in the NS + 3.0 V group as compared to the other groups, confirming that the H_2_-ECT is capable of activating cell apoptosis in tumor tissues. However, the underlying molecular and cellular mechanisms are unknown and require further study.

To confirm the vital role of H_2_ generation in the H_2_-ECT, we further investigated the effects of temperature and applied DC electric field on tumor growth. As shown in Fig. S13, since the temperature of the tumor remained basically unchanged before and after the H_2_-ECT treatment, the thermal effect in the H_2_-ECT process can be ignored. To check possible electrical field effect on tumor treatment, we also performed experiments under the same EC

conditions (3 V, 10 min) but using a silver needle with poorer EC H_2_ production capability instead of a stainless steel needle. As seen from Fig. S14, since the EC activity of silver (compared to Fe) is too low to effectively generate enough H_2_, the tumor suppression effect was obviously weakened. All these results fully illustrated the important role of sufficient H_2_ generation in the tumor therapy.

Next, to verify the generality of the method, we also applied the H_2_-ECT approach for the therapeutic treatment of another tumor model (MCF-7 breast cancer tumor), which was established by subcutaneously injecting MCF-7 breast cancer cells into the hind limb space of BALB/c nude mice (5 weeks old). Tumor-bearing mice were randomly divided into three groups: the blank, NS + 0 V and NS + 3.0 V groups. Similarly, the H_2_-ECT treatment under an applied voltage of 3 V was also applied for the MCF-7 tumor-bearing mice for 10 min twice a day for 3 days. After successive 3-day therapeutic periods and subsequent 12 days of observation, similar high-efficiency tumor suppression on the breast cancer was also achieved in the NS + 3.0 V group (Fig. S15). Our preliminary results implied the generality and rosy prospect of the H_2_-ECT for future minimally invasive treatments of cancers.

### *In vivo* biosafety of the H_2_-ECT

**Figure 4.**
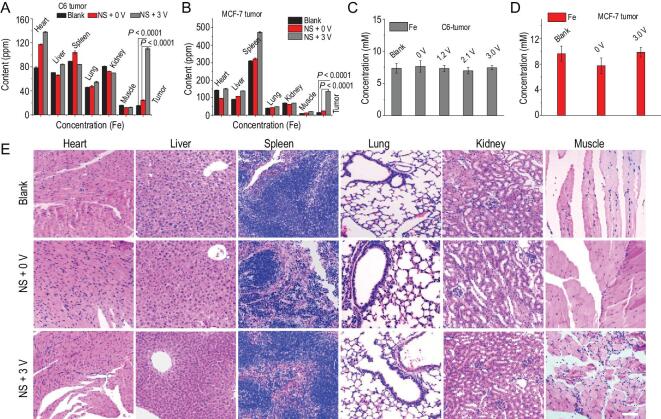
(A, B) Biodistributions of Fe element in tumor and main organs of tumor-bearing mice (C6 and MCF-7 cells) after the 15 days of treatment and observation. The error bars are standard deviations (*n* = 3). (C, D) The concentration of Fe element in the blood of C6 tumor-bearing mice of different groups. All the data are represented as mean ± SEM. *n* denotes the number of mice. *n* = 3, *P* < 0.0001 representing statistical significance, which is evaluated by Student's two-sided t-test with GraphPad Prism software compared to the control groups. (E) H&E staining images of major organs collected from C6 tumor-bearing mice in different groups after the H_2_-ECT treatment. Scale bar: 50 μm.

To evaluate the biosafety of the H_2_-ECT method, bio-distribution of iron element in the main organs for two kinds of tumor was determined using ICP-MS. As shown in Fig. 4A and B, iron element was found mainly accumulated into the tumor, without serious effects on other organs (heart, liver, spleen, lung, kidney and muscle). Moreover, the histopathological evaluation of major organs and hematology-related assays were carried out after the *in vivo* H_2_-ECT experiments. As shown in Fig. 4C and Fig. S16, major organs stained by H&E showed no significant pathological changes in the main organs between the control groups and the treatment groups. Meanwhile, the blood chemistry analyses were performed via checking the standard nine hematological biomarkers (see Figs S17 and S18), the results of which showed no distinct abnormality before and after the treatment for 15 days, indicating negligible side effects of the H_2_-ECT method. In addition, the microelements analyses (Fig. 4D and E, and Figs S19 and S20) showed no distinct microelement abnormality before and after the H_2_-ECT treatment, and the iron element in blood remained at a healthy level even after the treatment of H_2_-ECT at 3 V. All the above results showed the excellent biosafety of the H_2_-ECT method.

## DISCUSSION

Although H_2_ therapy with several successful clinical trials has been developed in the past decade, there remain huge challenges regarding its efficacy and methods. Compared to other biomedical applications, H_2_ therapy of cancer is rarely reported due to the lack of effective methods to control the production and release of enough H_2_ in the body. Due to the disadvantages of insufficient and uncontrollable release of H_2_ along with potential metabolic toxicity, current attempts to use nanomaterials as H_2_ carriers are still far from clinical oncotherapy.

In our study, we report a green and conceptually new *in vivo* H_2_ generation electrochemotherapy (H_2_-ECT) of tumors, by combined use of Chinese acupuncture Fe needle electrode and *in vivo* electrochemistry. The method solves the above problems by virtue of the acidic microenvironment of the tumor and *in vivo* electrochemical technology. The acupuncture electrodes inserted in the acidic tumor area, and working under the applied voltage of 3 V, produce effectively sufficient H_2_ to damage and kill cancer. The method is highly biosafe without an obvious poisoning effect, solving the difficult problems of material metabolism and biotoxicity in conventional therapeutic methods for clinical applications. By taking advantage of the puncture positioning, gas diffusion effect and self-protection mechanism of H_2_ generation, the H_2_-ECT method provides strong selectivity and targeting to effectively and precisely kill tumors, with minimal damage to healthy tissues. The effectiveness of the method has been demonstrated by *in vivo* treatment of glioma and breast cancers of diseased mice. More importantly, as the volume of the tumor tissue determines the total amount of hydrogen ions within the tumor, the larger tumors provide more hydrogen ions to produce enough hydrogen for effective treatment. Consequently, in principle, irrespective of the size of the tumor, the inherent volume effect of the method can solve the defect of insufficient hydrogen generation during the therapy process.

Although currently the method may not be valid for the treatment of non-solid tumors like lung cancer and liver cancer, our method is promising and reliable for the treatment of solid tumors. With the help of various advanced imaging techniques and cytopathology knowledge, we can accurately locate the tumor and carry out accurate treatment in future clinic applications, avoiding damage to normal tissue to the greatest extent. The developed H_2_-ECT is simple, safe, efficient and minimally invasive and therefore very promising for potential clinic applications to cure solid tumors. We believe that the H_2_-ECT method will also be used for the treatment of deep tumors with the help of accurate medical imaging technology in the future.

## METHODS

### Cell culture

The glioma cells (C6) and breast cancer cells (MCF-7) cells were obtained from the American Type Culture Collection (ATCC, USA). The MCF-7 and C6 cells were treated in Dulbecco's modified Eagle's medium (DMEM) supplemented with 10% fetal bovine serum (FBS), 100 U/mL penicillin, and100 μg/mL streptomycin at 37°C in a humidified atmosphere containing 5% CO_2_.

### Animal experiments

BALB/c nude mice (4 weeks old, 15 ± 0.2 g, male) were purchased from Beijing HFK Biotechnology Ltd (Beijing, China). Animal care and handing procedures were in agreement with the guidelines of the Regional Ethics Committee for Animal Experiments.

### Tumor models and *in vivo* H_2_-ECT treatments

The MCF-7 and C6 cells were cultured in DMEM supplemented with 10% FBS at 37°C in an atmosphere of 5% CO_2_. The MCF-7 and C6 cells were then collected and washed in cold PBS three times, respectively. After that 5 × 10^6^ cells/mL was dispersed into PBS and 50 μL of cell solution was injected into each mouse. The mice were used for further experiments when the tumor had grown to 8–10 mm in diameter. The mice were then divided into five groups at random: blank, needle stimulation (NS + 0 V) and NS + (1.2 V, 2.1 V and 3.0 V) and three mice were set as one group. For observation of the therapy effect of H_2_-ECT, C6 tumor-bearing mice were firstly anesthetized using intraperitoneal injection of chloral hydrate solution (10 wt%). The purpose of anesthesia was to make the mice unconscious and eliminate pain, and keep them still during the H_2_-ECT therapy. Then the tumor sites were disinfected using 75% alcohol and subsequently two acupuncture electrodes were inserted into the tumor lump. The acupuncture electrode was inserted into the central part of the tumor tissue, which can avoid damage to the normal tissue. From an electrochemical point of view, the distance between the two electrodes will affect the efficiency of H_2_ generation *in vivo* to some extent. Therefore, in order to avoid short circuits and excessive electrochemical resistance in tumor tissues, and thus achieve the effectiveness and repeatability of the treatment, we chose the optimal distance (between two electrodes) of ∼3–5 mm for the treatment in this study. Finally, the two electrodes were connected to the positive and negative poles of the voltage regulator, respectively, and single H_2_-ECT treatment carried out under the various applied voltages from 0 to 3.0 V for 10 min. Each tumor-bearing mouse was treated with H_2_-ECT twice per day for 3 days and then observed for another 12 days. To avoid excessive injury to normal tissue, the negative pole should be inserted into the center of the tumor bulk. The insertion depth of the acupuncture electrode depends on the depth and thickness of the tumor and the outside part of the acupuncture electrodes was insulated to avoid unnecessary injury to normal tissue. Although acupuncture Fe electrodes can be reused for H_2_-ECT, since they are very cheap (less than $0.1) and readily available, we used them only once per treatment to avoid infection and contamination between mice.

The tumor size was measured using the digital caliper in two dimensions and the tumor volume was calculated by the following equation: tumor volume = (length × width^2^)/2. Meanwhile, the body weight changes of mice were recorded every day to estimate the efficacy of physical recovery. On the 16th day, all the mice were dissected to collect their organs (heart, liver, lung, kidney and spleen) fixed by paraformaldehyde (4 wt%) for pathological analysis. The tumors were weighed to assess therapy efficacy of different experimental groups.

## Supplementary Material

nwz199_Supplemental_File
