# Supplementary material for: A green, efficient and precise hydrogen therapy of cancer based on in vivo electrochemistry
Source: Natl Sci Rev. 2019 Dec 5;7(3):660–70. doi: 10.1093/nsr/nwz199 (PMC8288856; doi:10.1093/nsr/nwz199)
Supplement: nwz199_Supplemental_File [file nwz199_supplemental_file.pdf]

# Supplementary Materials for

## A green, efficient and precise hydrogen therapy of cancer based on in-vivo electrochemistry

Guohua Qi, Bo Wang, Xiangfu Song, Haijuan Li, Yongdong Jin*

* Corresponding author. Email: ydjin@ciac.ac.cn

**Materials**

The simulated body fluid (SBF) was purchased from the Yuan Ye Biotechnology Co., Ltd (Shanghai). $KH_2PO_4$, $Na_2HPO_4$, NaCl and KCl were bought from Shanghai Aladdin Reagent Co. The methylthionine chloride, 3-(4,5-dimethyl-2-thiazolyl)-2,5-diphenyl-2-H-tetrazolium bromide (MTT), paraformaldehyde, chloral hydrate and green vitriol were purchased from Aladdin. The dimethylsulfoxide (DMSO) was purchased from KeyGen Biotech. Co. Ltd. The Dulbecco's modified Eagle's medium (DMEM), fetal bovine serum (FBS), 0.25% trypsin/2.2 mM EDTA solution and antibiotic solution contained 200 units $mL^{-1}$ penicillin and 200 units $mL^{-1}$ streptomycin were bought from Biological Industries. The acupuncture needles (diameter: 0.35 mm; length: 40 mm) were obtained from Medical Supplies Factory Co., Ltd (Suzhou). Calcein-AM and Propidium Iodide (PI) double staining kit were purchased from Sigma-Aldrich (USA). Haematine, eosin and TUNEL cell apoptosis detection kit were obtained from ThermoFisher.

## Instruments

The ESCALAB-MKII spectrometer (VG Co., UK) with Al KR X-ray radiation as the X-ray source for excitation was applied for conducting the X-ray photoelectron spectroscopy (XPS). Fluke infrared thermometer (TiS40) was performed for infrared imaging. The enzyme-labelled meter was purchased from Tecan. Stabilized voltage supply (HY3005B) which purchased from HuaYi Instrument Co., Ltd. was used for $H_2$-ECT experiments. The biodistribution of Fe in vivo was detected using the ICP-MS (Thermo Scientific Icap 6300).

## Electrochemical experiments

The electrochemical experiments of simulated body fluid (SBF) under the different pH values were tested in a standard three electrode system on a CHI 660E electrochemical workstation at room temperature. The Ag/AgCl (KCl saturated) and the acupuncture electrode whose main material is stainless steel were selected as the reference electrode and counter electrode, respectively. We also chose the acupuncture electrodeas working electrode. The polarization curves of SBF solution with pH value of 5.5, 6.0, 6.5, 7.0 and 7.5 were respectively detected. Simultaneously, polarization curves of tissue samples of pig muscle under the different pH values were also detected at the same conditions. Before the measurement, the electrolyte was purged with $N_2$ to remove oxygen in electrolyte for 20 min. The polarization curves were performed with

a scan rate of 2 mV $s^{-1}$. The current density was normalized to the geometrical area of the electrode.

## Cell viability assay

The cell viability was detected through the standardized MTT assay. Typically, the MCF-7 and C6 cells were seeded on the 96-well microtiter plates and incubated for 24 h at 37 °C. Then the cells were washed using the cold PBS by three times. After that 10 μL of different concentrations of ferrisulfas (from 5 μM to 50 mM) was added to each well supplemented with 90 μL of complete medium for 24 h at 37 °C. Then the cells were cleaned by the cold PBS by three times. Finally, 5 μL of MTT solution (5 mg/mL) was added into each well and was treated at 37 °C for another 4 h in the $CO_2$ incubator. The 150 μL of DMSO after removing the supernatant medium was added into each well after the reaction to dissolve the formed purple formazan crystals. The absorbance of the wells was tested on a microplate reader with the measurement wavelength of 570 nm. Cells incubated in the absence of ferrisulfas were used as a control.

### Blood chemistry analysis

At 16$^{th}$ day, the blood of the tumor-bearing mice in different groups was drawn from the eye socket. The healthy mice were set as the control group. Part of each blood sample was used for trace microelement analysis and the rest was applied for routine analysis of blood by the Automatic Blood Analyzer using protocols approved by Jilin ADICON Clinical Laboratories. Inc.

### Histological Examination

The organs and tumor from each group were collected at the 16$^{th}$ day, fixed, sectioned, and stained following the standard protocol for H&E staining. The TUNEL assays of tumor tissue were executed by the instruction of in-situ Cell Death Detection kit (Roche). The images of different groups were observed using the fluorescence microscope (BX51T-PHD-J11).

### X-ray CT Imaging

X-ray CT imaging experiments were carried on a Philips 256-slice CT Scanner (Philips Medical System). When the tumor volumes were close to 200 $mm^3$, the tumor-bearing mice were treated with $H_2$-ECT of different groups. The CT images were obtained at various voltages after the $H_2$-ECT (Blank, NS+0 V, NS+1.2 V, NS+2.1 V and NS+3.0 V). The imaging parameters were set as:

thickness, 0.9 mm; 120 kVp, 300 mA; pitch, 0.99; field ofview, 350 mm; gantry rotation time, 0.5 s; table speed, 158.9 mm $s^{-1}$. Thin-section axial images were reformed to coronal images by a computational technique referred to as multiplanar-reconstruction.

**In Vivo MR imaging**

To provide evidence of gas production after the $H_2$-ECT, the MR imaging of C6 tumor-bearing mice were performed at different groups. Firstly, the C6 tumor-bearing mice were anaesthetized by intraperitoneal injection of chloral hydrate solution (10 wt%) and then the C6 tumor-bearing mice were treated with $H_2$-ECT at different voltages for 10 min. The $T_1$-weighted images were acquired using a 1.5 T human clinical scanner.

**Biodistribution of Fe in Vivo**

At the 16$^{th}$ day, all the C6 tumor-bearing mice (n=3) including control and experimental groups (NS and NS+3.0 V) were dissected to collect the organs of heart, liver, spleen, lung, kidney, muscle and tumor. The organs were wet weighted and then dissolved in digesting aqua regia ($HNO_3$: HCl = 1:3) for 48 h. The amounts of Fe in different samples were analyzed through ICP-AES.

**Supplementary Figure S1**

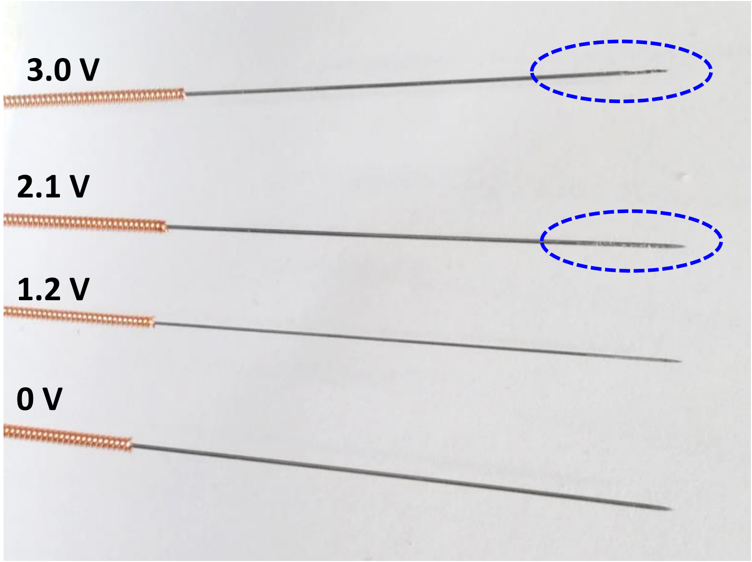


**Figure S1.** The picture of the anode acupuncture Fe electrodes after the electrolytic reaction in the SBF (pH=6.0) under the different voltages (0, 1.2, 2.1 and 3.0 V).

**Supplementary Figure S2**

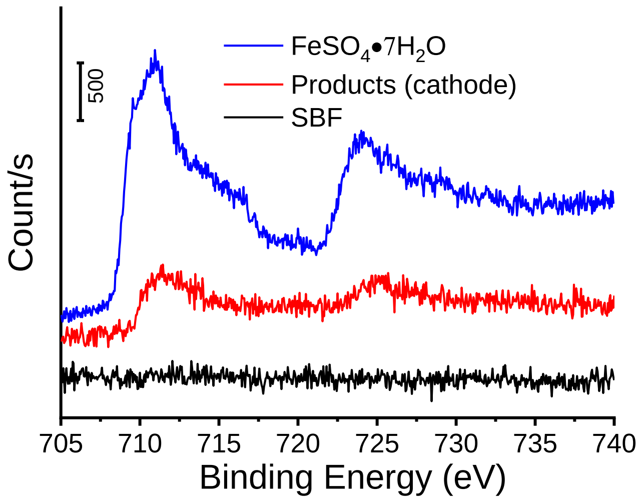


**Figure S2.** The typical XPS spectra of Fe recorded from the SBF, the products of anode after the $H_2$-ECT for 10 min under 3.0 V, and the standard $FeSO_4{\cdot}7H_2O$ compound, respectively.

**Supplementary Figure S3**

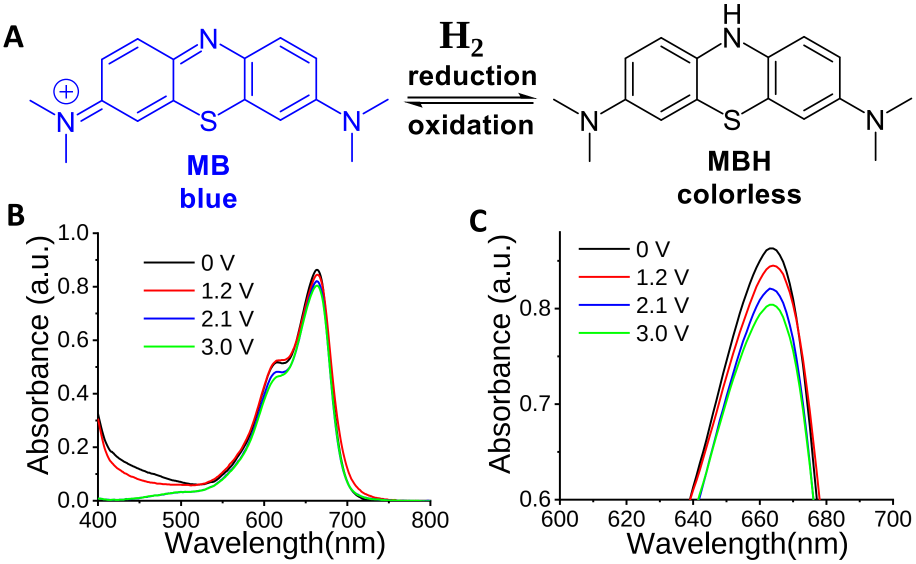


**Figure S3.** (**A)** The $H_2$ detection mechanism of the MB probe based on the color change during the redox reaction. (**B**) Absorption spectra and (**C**) locally enlarged spectrogram of MB at different concentrations of $H_2$ produced under the different voltages.

**Supplementary Figure S4**

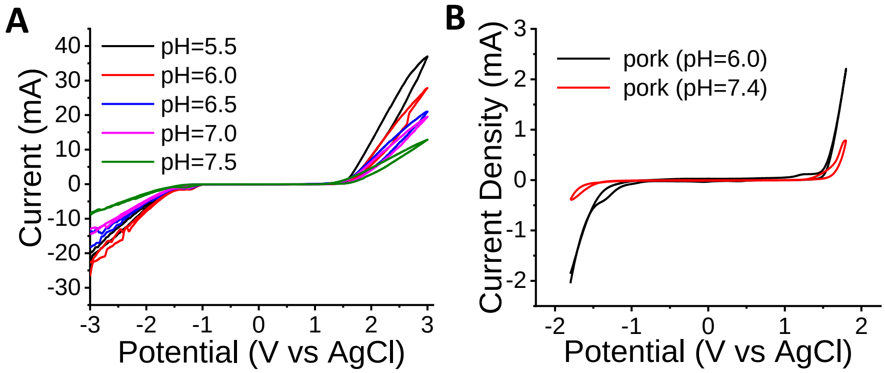


**Figure S4.** (**A**) The cyclic voltammograms of the acupuncture Fe electrode recorded in the SBF of different pH values (5.5, 6.0, 6.5, 7.0, 7.5). (**B**) The cyclic voltammograms of the acupuncture Fe electrode recorded in two fresh pork tissues with different simulated pH values.

**Supplementary Figure S5**

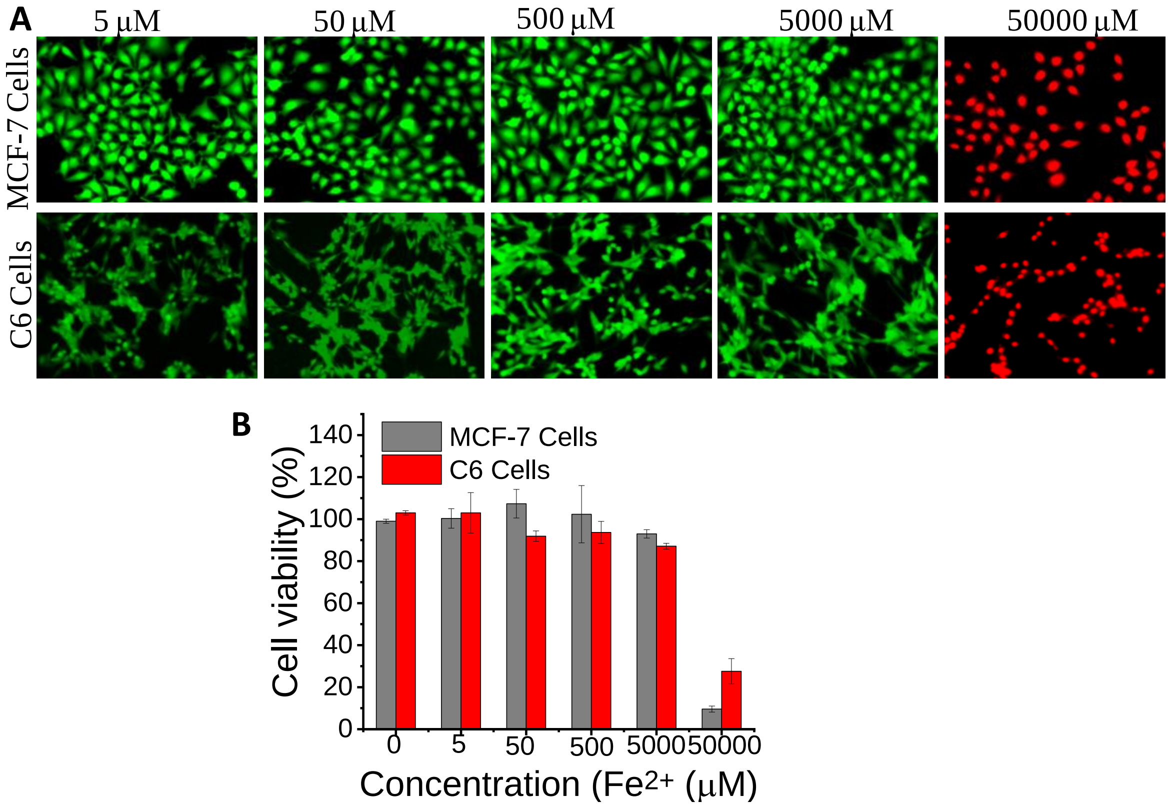


**Figure S5.** (**A**) The Live/dead cell staining and (**B**) cell viability of MCF-7 and C6 cells incubated with the different concentrations of $Fe^{2+}$ ($FeSO_4 \cdot 7H_2O$) for 24 h detected using the fluorescence imaging and MTT assay.

**Supplementary Figure S6**

-

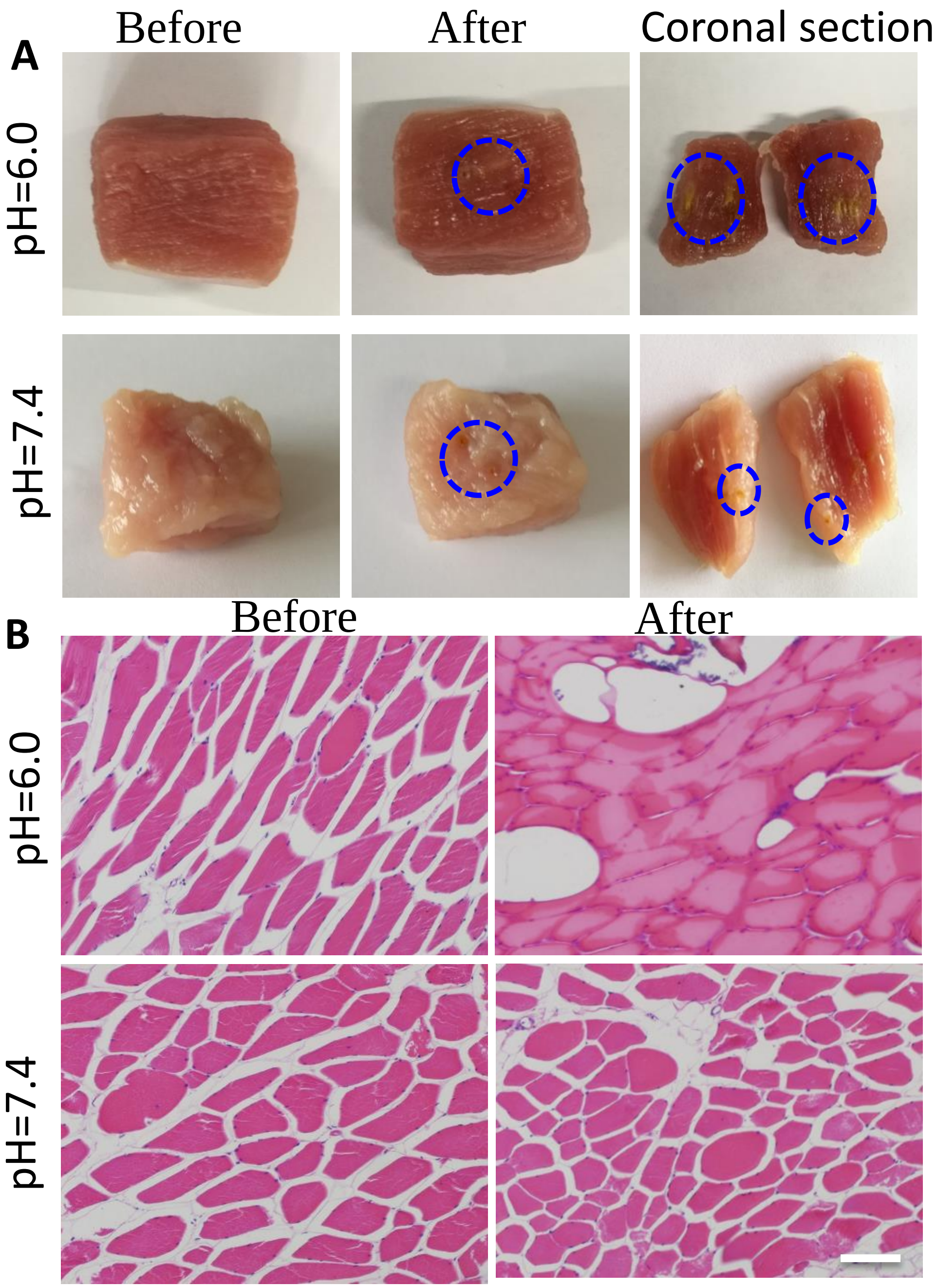


**Figure S6.** (**A**) The pictures of the fresh pork after immersing into the SBF of two simulated pH (6.0 and 7.4) for 24 h, followed by the $H_2$-ECT treatment at 3V for 10 min. (**B**) H&E staining images of fresh pork with different pH (6.0 and 7.4) before and after the $H_2$-ECT for 10 min at 3 V. The scale bar is 50 μm.

## Supplementary Figure S7

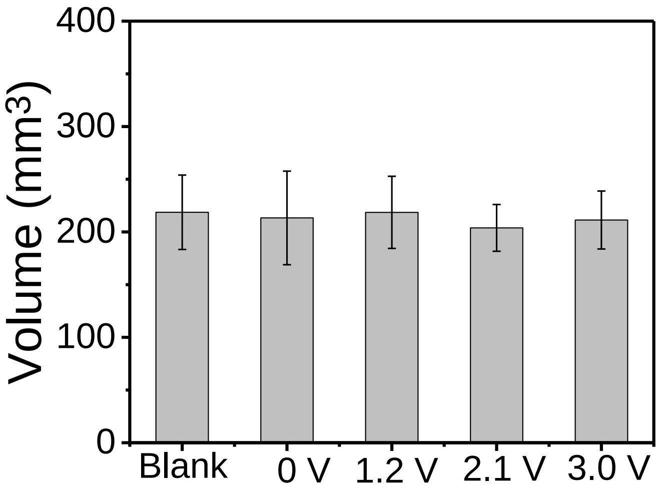


**Figure S7.** The tumor volume of C6 tumor-bearing Balb/c mouse under different groups (Blank, NS+0 V, NS+1.2 V, NS+2.1 V and NS+3.0 V) before $H_2$-ECT. Each group was averaged from three mice.

## Supplementary Figure S8

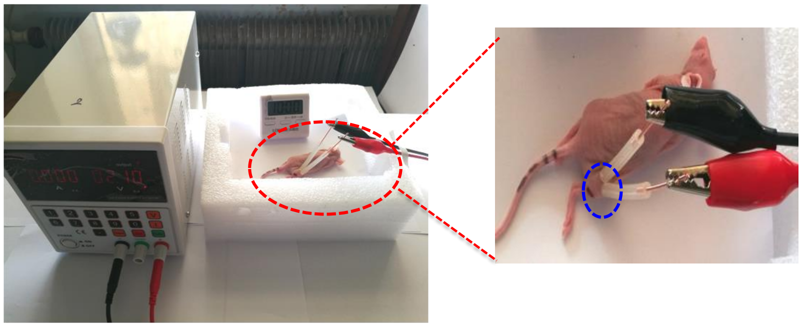

**Figure S8.** The experimental platform used for the $H_2$-ECT treatment of tumor in vivo.

## Supplementary Figure S9

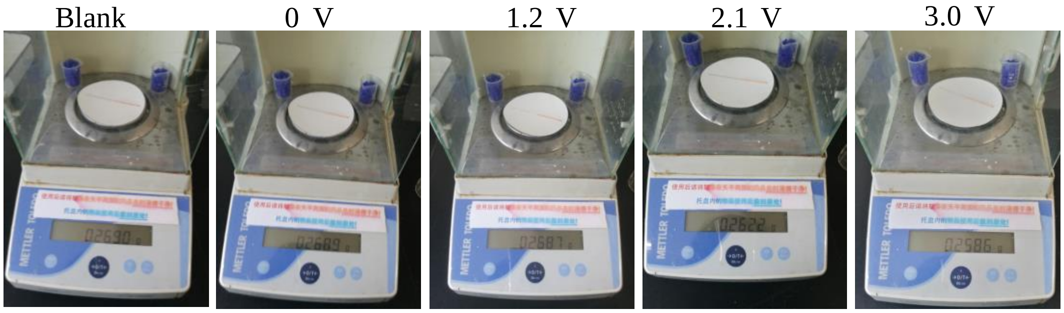


**Figure S9.** The photograph of an acupuncture Fe electrode (anode) showing its weight loss after the $H_2$-ECT treatment of tumor in vivo.

**Electrochemical estimation of volume of $H_2$ generated during one $H_2$-ECT treatment (at 3 V, for 10 min), by using the Faraday's law of electrolysis, as follows:**

Firstly, the integral area of average current curve with time (**Extended Data Fig. 10**) was calculated about 63.39

$I=\frac{dQ}{dt}$; $Q=\int_0^t Idt=63.39$

Q= z F n (Fe) =2×96500×n (Fe)=63.39

m (Fe) $=\frac{Q}{zF}M_{Fe}$

PV=nRT ; V=nRT/P=m/M(Fe)×RT/P

V ($H_2$) $=\frac{QRT}{zFP}$=63.39×8.314×298/(2×96500×101325)=8.03 mL (under standard atmospheric pressure, $P^{\odot}$)

Where R=8.314J.mol; T=298 K, z=2, F=96500 C/$mol^{-1}$; P=101.325kPa

## Supplementary Figure S10

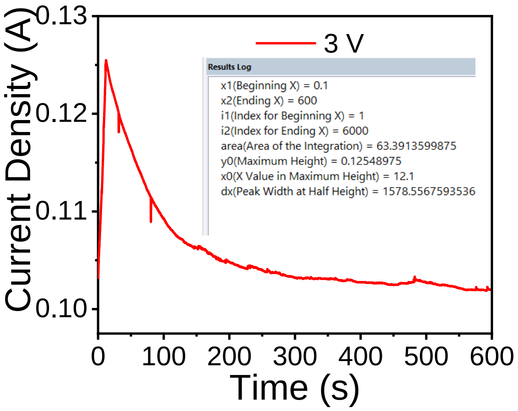


**Figure S10.** The average current curve of the acupuncture Fe electrode with time during the $H_2$-ECT under 3 V for 10 min (n=4).

## Supplementary Figure S11

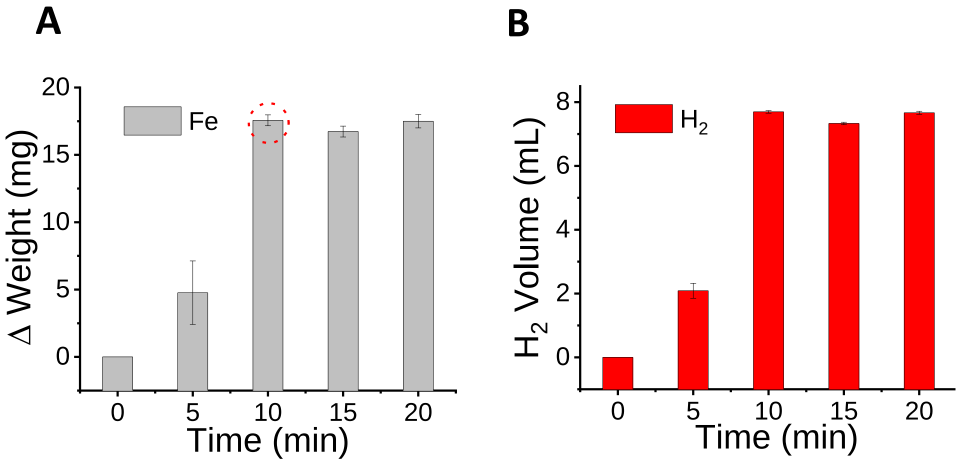


**Figure S11.** The electrogravimetric analysis of the acupuncture Fe electrode and the corresponding volume change of hydrogen with the time during the $H_2$-ECT treatment (at 3 V) for the C6 tumor-bearing Balb/c mice.

## Supplementary Figure S12

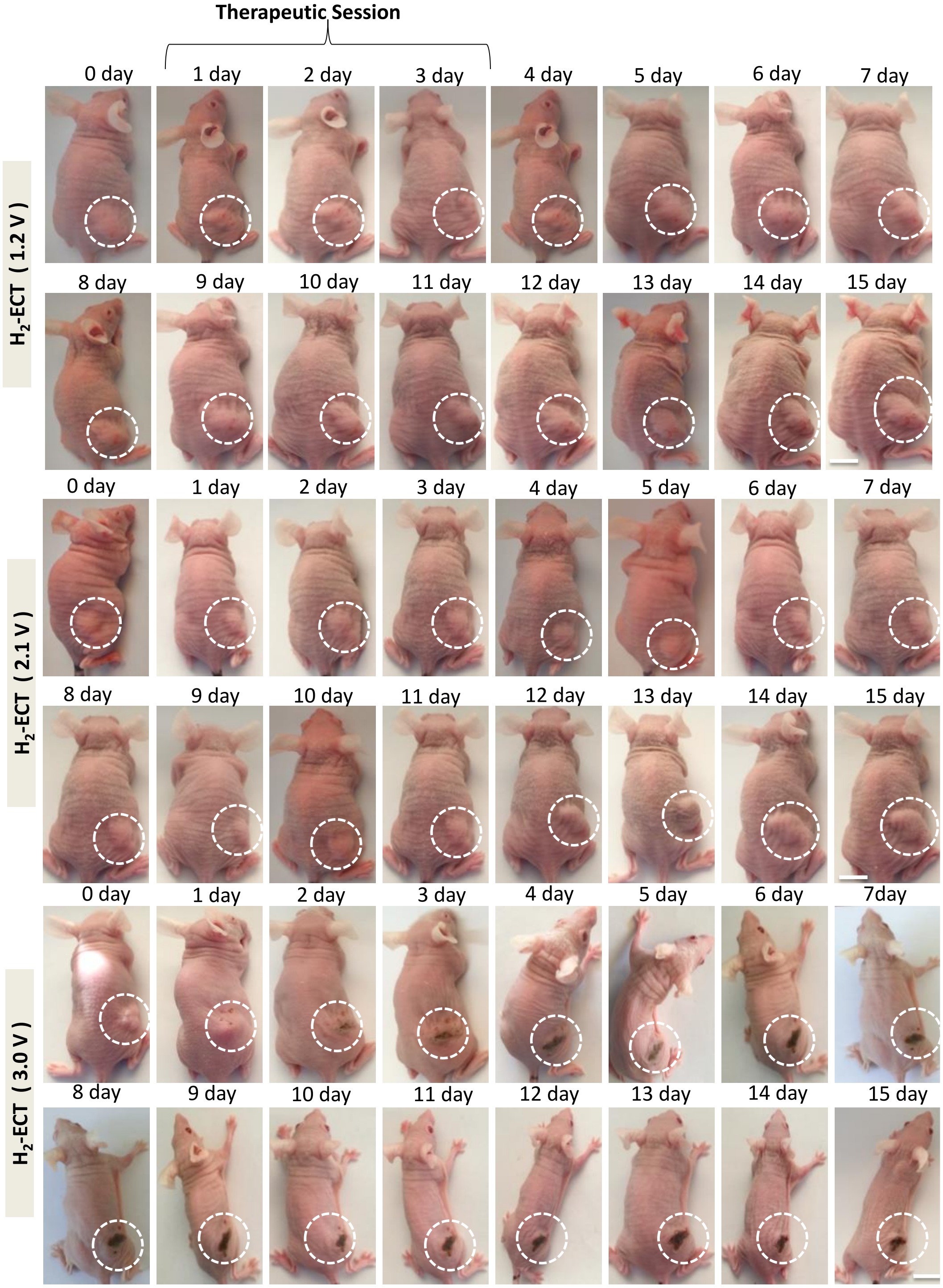


**Figure S12.** Photos of a C6 tumor-bearing Balb/c mouse during the whole 15-day $H_2$-ECT treatment and recovery process under the different voltages (1.2, 2.1 and 3.0 V). Scale bar: 1.5 cm.

**Supplementary Figure S13**

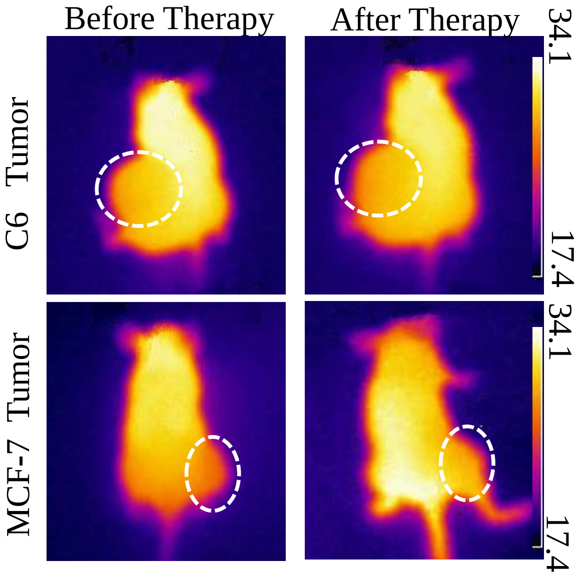


**Figure S13.** The near infrared thermal imaging of the C6 tumor-bearing and MCF-7 tumor-bearing Balb/c mice, respectively, before and immediately after the $H_2$-ECT treatment (at 3 V) for 10 min.

**Supplementary Figure S14**

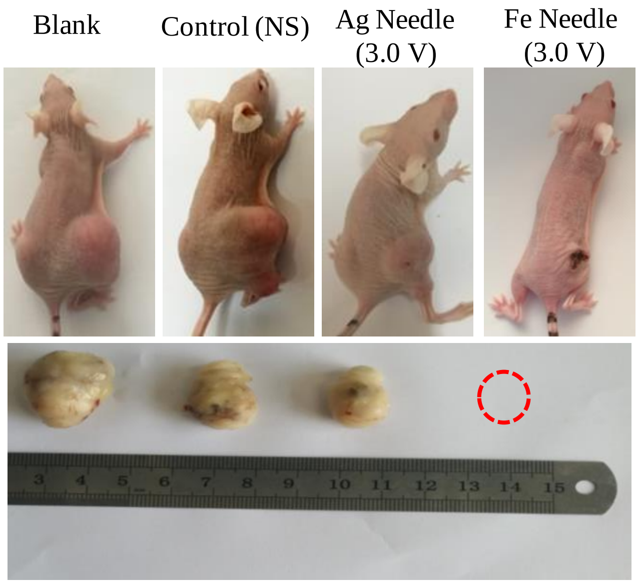


**Figure S14.** The comparison of the tested mice and tumors after the $H_2$-ECT treatment under three different conditions (blank, Control (NS, Fe needle), NS+3.0 V (Ag needle), NS+3.0 V (Fe needle) for 10 min, twice a day for 3 days, and a 12-day recovery period.

## Supplementary Figure S15

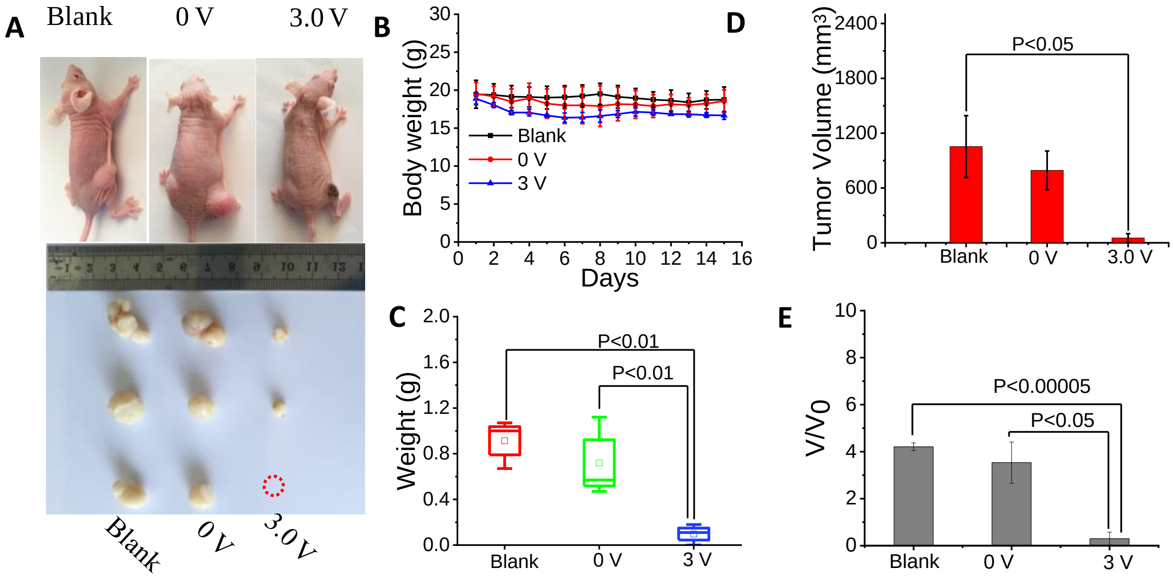


**Figure S15. (A)** Typical photographs of three groups of MCF-7 tumor-bearing mice and tumors after the 15-day $H_2$-ECT treatment and observation period. Red circles indicate the elimination of tumor. (**B**) The body weights of the MCF-7 tumor-bearing nude mice with three different groups and recorded every day. (**C**) Tumor weight and (**D**) tumor volume obtained by dissection, and relative tumor volume (**E**) (n=3). Statistical significance is evaluated by Student's two-sided t-test compared to the control group. All the Data are represented as mean ± SEM. n denotes mice.

**Supplementary Figure S16**

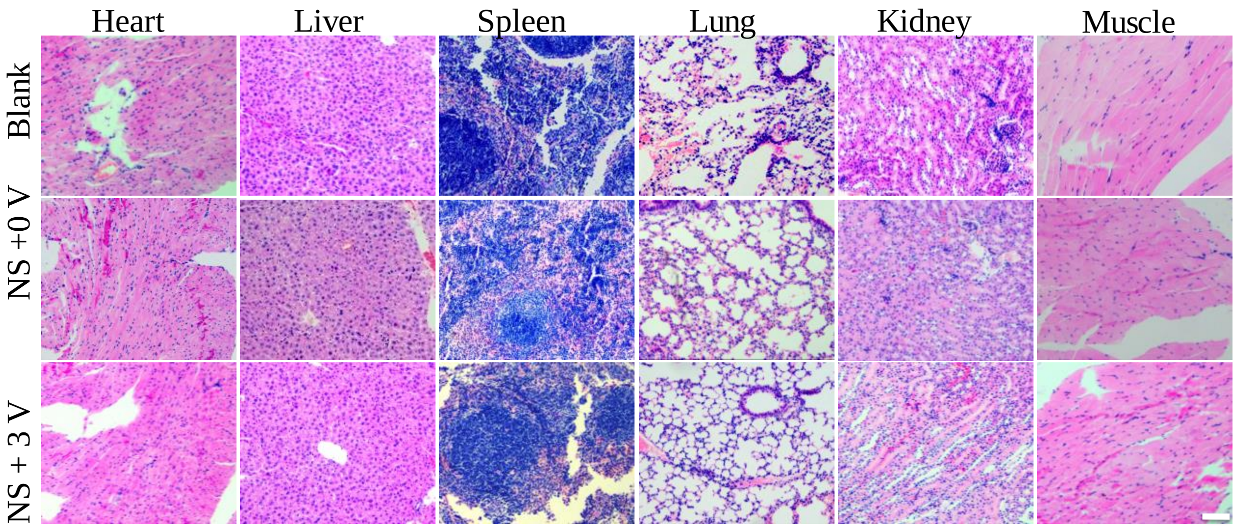


**Figure S16.** H&E staining images of major organs collected from mice of breast cancer tumor in three different groups. The scale bar is 50 μm.

**Supplementary Figure S17**

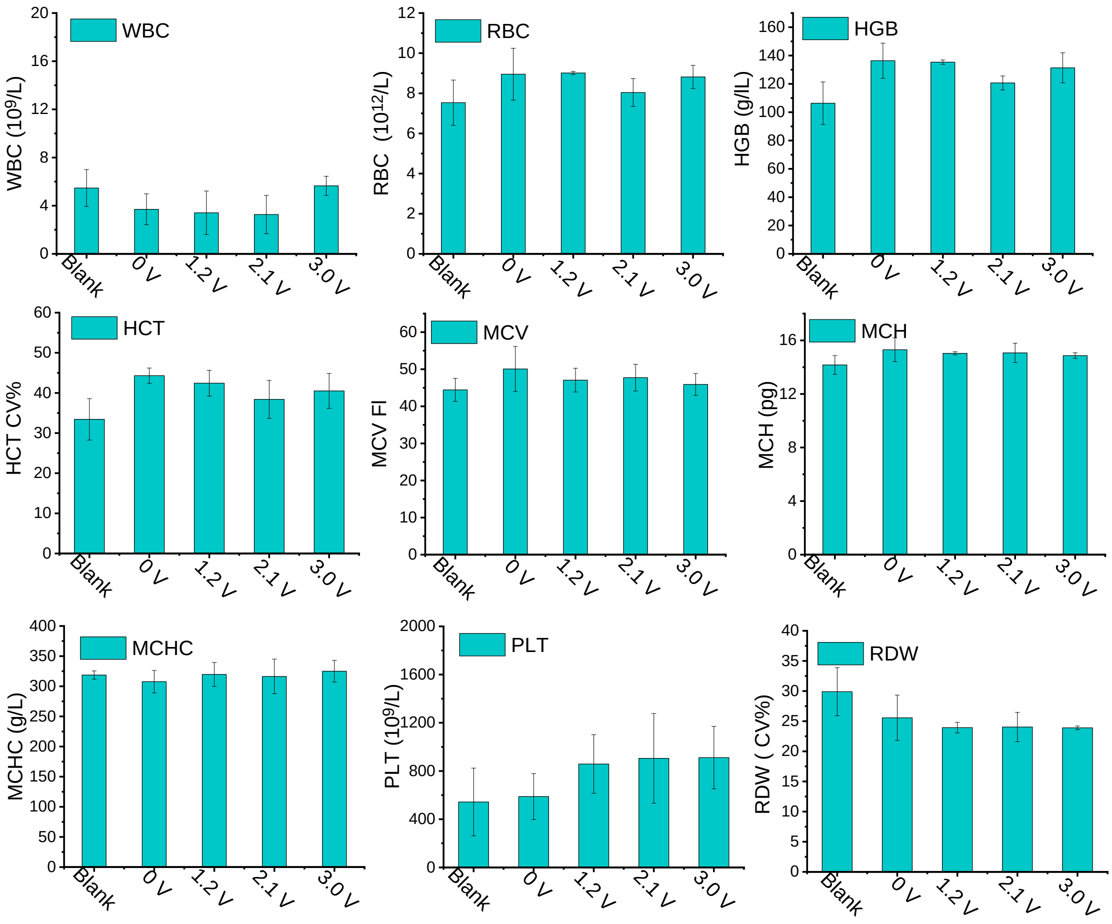


**Figure S17.** Haematology data of the C6 tumor-bearing mice in different treatment schemes as compared to healthy mice.

## Supplementary Figure S18

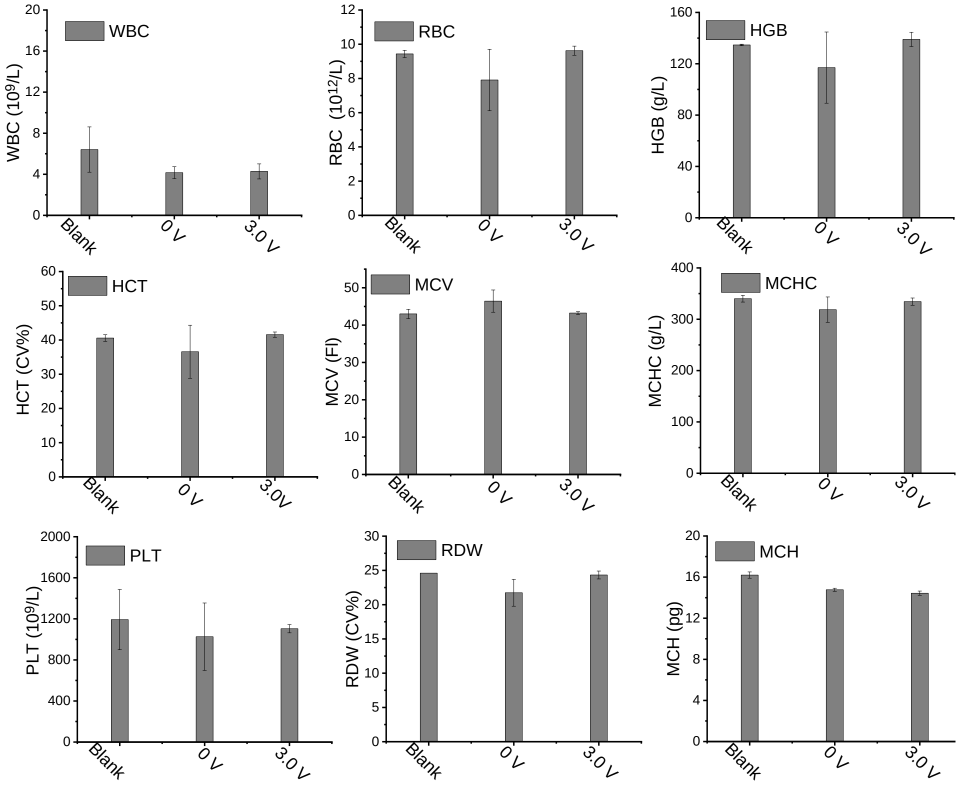


**Figure S18.** Haematology data of the MCF-7 tumor-bearing mice in different treatment schemes as compared to healthy mice.

**Supplementary Figure S19**

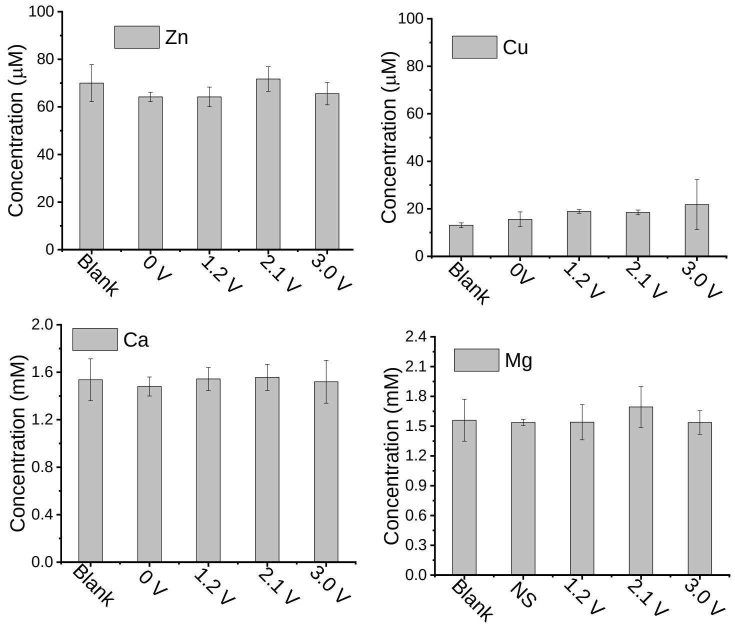


**Figure S19.** The microelement concentration assays in the C6 tumor-bearing mice blood of different groups.

**Supplementary Figure S20**

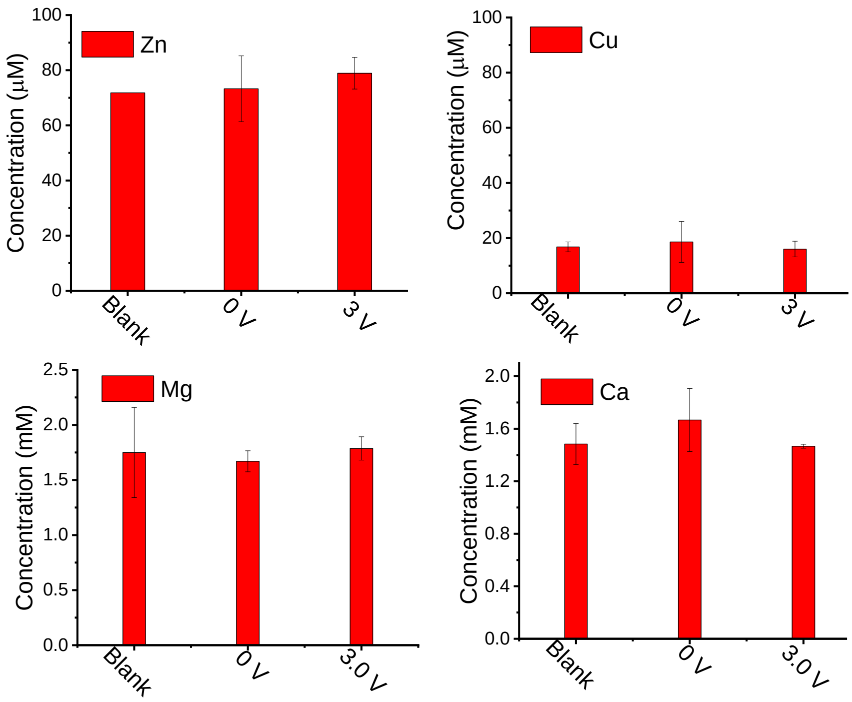


**Figure S20.** The microelement concentration assays in the MCF-7 tumor-bearing mice blood of different groups as compared to healthy mice.
